# High Seroprevalence of Anti-SARS-CoV-2 IgM/IgG among Inhabitants of Sakaka City, Aljouf, Saudi Arabia

**DOI:** 10.3390/vaccines11010026

**Published:** 2022-12-22

**Authors:** Ahmed E. Taha, Abdulrahman A. Alduraywish, Abdulrahman H. Almaeen, Tarek H. El-Metwally, Mohammad Alayyaf, Ayesha Mallick, Mohamed Abouelkheir

**Affiliations:** 1 Microbiology and Immunology Unit, Department of Pathology, College of Medicine, Jouf University, Sakaka 72388, Saudi Arabia; 2 Medical Microbiology and Immunology Department, Faculty of Medicine, Mansoura University, Mansoura 35516, Egypt; 3 Department of Internal Medicine, College of Medicine, Jouf University, Sakaka 72388, Saudi Arabia; 4 Department of Pathology, Pathology Division, College of Medicine, Jouf University, Sakaka 72388, Saudi Arabia; 5 Department of Pathology, Biochemistry Division, College of Medicine, Jouf University, Sakaka 72388, Saudi Arabia; 6 Department of Medical Biochemistry, Faculty of Medicine, Assiut University, Assiut 71517, Egypt; 7 Consultant Histopathologist & Nephropathologist, Medical Lab Director, Prince Mutaib Bin Abdulaziz Hospital, Sakaka 72388, Saudi Arabia; 8 Department of Community & Family Medicine, College of Medicine, Jouf University, Sakaka 72388, Saudi Arabia; 9 Department of Pharmacology and Therapeutics, College of Medicine, Jouf University, Sakaka 72388, Saudi Arabia; 10 Pharmacology Department, Faculty of Medicine, Mansoura University, Mansoura 35516, Egypt

**Keywords:** antibodies, COVID-19, immunity, prevalence, SARS-CoV-2, vaccination

## Abstract

(1) Backgrounds and Objectives*:* The global battle to contain the severe acute respiratory syndrome-coronavirus-2 (SARS-CoV-2) is still ongoing. This cross-sectional study aimed to detect the seroprevalence of anti-SARS-CoV-2 IgM/IgG among previously symptomatic/asymptomatic and vaccinated/unvaccinated inhabitants of Sakaka City, Aljouf, Saudi Arabia. (2) Methods: Blood samples of 400 participants were tested for the presence of anti-SARS-CoV-2 IgM/IgG using colloidal gold immuno-chromatography lateral flow immunoassay cards. (3) Results: The prevalence of anti-SARS-CoV-2 IgM and IgG positivity was 45.8% and 42.3%, respectively. Statistically significant correlations (*p* < 0.05) were found between the previous RT-PCR testing for SARS-CoV-2-RNA and positivity for IgM and/or IgG. The highest seroprevalence of IgM and IgG were detected among smokers, participants aged ≥40 years, and patients with chronic diseases. Although most of the participants (58.5%) did not previously experience COVID-19 like symptoms, the anti-SARS-CoV-2 IgM and IgG seropositivity amongst them was 49.1% and 25.6%, respectively, with higher seroprevalence among males than females. At the time of the study, the SARS-CoV-2 vaccination rate at our locality in Saudi Arabia was 43.8% with statistically significant correlation (*p* < 0.001) between being vaccinated and anti-SARS-CoV-2 IgM and/or IgG positivity, with more positivity after receiving the second vaccine dose. (4) Conclusions: Public assessment reflects the real scale of the disease exposure among the community and helps in identifying the asymptomatic carriers that constitute a major problem for controlling the SARS-CoV-2. To limit the spread of the virus, rigorous implementation of large-scale SARS-CoV-2 vaccination and anti-SARS-CoV-2 serological testing strategies should be empowered.

## 1. Introduction

The global battle to contain the coronavirus disease-2019 (COVID-19) pandemic, caused by the severe acute respiratory syndrome-coronavirus-2 (SARS-CoV-2), is still ongoing, and there are many lessons that should be learned from the previous infections, outbreaks, epidemics, and pandemics [1]. The ‘shield immunity’ against certain infectious diseases depends primarily on the duration and strength of immunity acquired after being infected [2]. Several studies reported variable durations of antibody response after infection with SARS-CoV or Middle East respiratory syndrome (MERS-CoV). Circulating anti-SARS-CoV and anti-MERS-CoV immunoglobulins persisted for more than 1–2 years [3,4,5,6,7]. There are speculations that previous exposure to viral strains of the same family could offer cross-immunity and protection from infection among its members [8]. It was reported that anti-MERS-CoV immunoglobulins, which were detected in many asymptomatic persons exposed to dromedaries and camels and considered as a semi-immunity against similar strains of the virus, might explain the geographical discrepancies in the reported severity of cases [9,10]. The percentage of asymptomatic carriers differs widely across infectious diseases, ranging from 90–95% for poliovirus, 32% for norovirus infections, and down to 8% for measles [11,12,13].

Although SARS-CoV-2 originates from the same family as the SARS-CoV and MERS-CoV viruses, its high rate of spread and pathogenesis are not fully understood. This may be due to the higher rates of SARS-CoV-2 replication, human-to-human transmission, and asymptomatic carriage than other coronaviruses [14,15]. The SARS-CoV-2 infected persons can be either asymptomatic (mostly in children and young adults) or symptomatic with mild, moderate, or severe presentation (mostly in patients with comorbidities) [16,17]. The COVID-19 severity varies due to different demographic characteristics, comorbidities, and immune responses among different populations [18,19]. The asymptomatic carriers are dangerous sources for the transmission of this fatal virus and represent an infection control challenge. SARS-CoV-2 infected persons can spread the virus before and during the symptomatic course of the disease and even during the recovery period [20].

Specific humoral immune response against SARS-CoV-2 can be induced in most symptomatic cases and asymptomatic carriers, where the primary immune response (IgM) appears 3 to 10 days after infection, followed by the secondary immune response (IgG) two weeks after infection then lasts for months [21,22]. Early testing strategies are central to succeed in the SARS-CoV-2 infection control policy. Molecular detection of SARS-CoV-2-RNA by RT-PCR is the golden test to diagnose persons who are currently infected. Serological tests can detect individuals who are exposed to the virus and consequently, they developed antibodies. Accordingly, large scale serological tests may be used to reflect the real spread of the virus in the community. Lateral flow immunoassay (LFIA), chemiluminescence immunoassay (CLIA), and enzyme-linked immunosorbent assay (ELISA) are among the most popular serological tests to detect the anti-SARS-CoV-2 immunoglobulins. Depending on the manufacturers, LFIA has an average specificity and sensitivity values > 90% [23].

Besides the infection prevention and control strategies, the international collaboration to control the SARS-CoV-2 pandemic resulted in rapid development of many SARS-CoV-2 vaccines [24,25]. Worldwide, as of 15 November 2022, the number of COVID-19 confirmed cases reported to the WHO was 632,533,408 including 6,592,320 deaths, and a total of 12,885,748,541 SARS-CoV-2 vaccine doses have been administered as of 8 November 2022 [26]. In Saudi Arabia, there have been 824,640 COVID-19 confirmed cases, including 9435 deaths, reported to the WHO as of 15 November 2022, and a total of 67,979,420 SARS-CoV-2 vaccine doses have been administered as of 26 October 2022 [27]. SARS-CoV-2 vaccines induce both cellular and humoral immune responses that include vaccine-induced neutralizing antibodies to the receptor-binding domain of the SARS-CoV-2 S protein, which are near-completely protective in animal studies [28].

Understanding the humoral immune response to SARSCoV-2 and its vaccines is crucial for optimization of the anti-SARSCoV-2 community immunization programs. There is a gap in the knowledge about the humoral immunity of SARS-CoV-2 infected/vaccinated persons in Sakaka, Aljouf, Saudi Arabia. We aimed to assess the seroprevalence of anti-SARS-CoV-2 IgM/IgG among previously symptomatic/asymptomatic and vaccinated/unvaccinated persons and correlate the results to relevant socio-demographics, anthropometrics, lifestyle, and medical factors.

## 2. Methodology

### 2.1. Study Design

A cross-sectional study was conducted on the inhabitants of Sakaka city, Aljouf, Saudi Arabia who agreed to participate, over a period of 4 months starting from July to October 2021. The sample size was calculated by using the online (Roasoft) sample size calculator (http://www.raosoft.com/samplesize.html (accessed on 10 April 2021) with margin of error 5%, response distribution 50%, and confidence level 95% for the total Sakaka population of 250,000. In total, 400 participants were included in this study.

Employees, workers, students, and staff of Jouf University, their relatives, and visitors to different city malls and the primary healthcare centers (through the collaboration of healthcare providers) were voluntarily recruited sequentially at their first appearance until the required sample size was met. Each participant was educated about the study and invited to sign an informed consent form. Relevant data were collected using a predesigned data collection proforma, then a drop of blood was obtained for testing.

### 2.2. Data Collection

The collected data inquired about socio-demographics, anthropometrics, and COVID-19 history of the participants, including: Age, gender, body mass index (calculated as weight in kg/height in m^2^), life style (smoking, physical activity, diet/beverages and herbal preferences), comorbidities (hypertension, diabetes and its type, liver, heart and kidney diseases, cancer, gastroesophageal reflux disease, autoimmune disease, chronic inflammatory diseases, etc.), type and duration of the current medications (anti-diabetic, antihypertensive, antibiotic, anti-inflammatory/immunosuppressive, blood thinners, etc.), previous PCR testing for SARS-CoV-2-RNA and its result, previous experience with symptoms similar to those of COVID-19 disease, previous contact with a person who was RT-PCR-positive for SARS-CoV-2-RNA, previous contact with a person who was suffering from symptoms similar to those of COVID-19 but he/she did not have RT-PCR testing or his/her RT-PCR test for SARS-CoV-2-RNA was negative, and SARS-CoV-2 vaccination history.

### 2.3. Blood Samples Collection

After skin antisepsis with an alcohol-based swab, a small finger prick drop of capillary blood was dispensed into the assay detection card directly or using disposable micropipette. Bio-wastes were collected in color coded bags and sharp boxes for proper disposal.

### 2.4. Antibodies Testing Technique

Anti-SARS-CoV-2 IgM and IgG were detected in the capillary blood droplet using colloidal gold immuno-chromatography LFIA cards with specificity and sensitivity values > 90% (SunLong Biotech Co., LTD, Hangzhou, China). The assay uses recombinant antigens LQPELDSFKEELDKYFKNHTSPDVD from the spike protein of SARS-CoV-2 conjugated with a nucleoprotein and immobilized on specific particles. After applying the sample into the sample well of the test cassette, it flows laterally across the pad. At the test line, the anti-SARS-CoV-2 immunoglobulins, if present in the sample, react with the SARS-CoV-2-antigen-coated particles in the test strip, thus, the test line became visible indicating positive sample. Visualizing a control line, at which nanoparticle-linked antibodies bind to the specific antibodies, verifies the accuracy of the test. The interpretations were positive (when both lines were visible), negative (when only the control line was visible), or invalid (only the test line was visible) results. In addition, the invisibility of both lines indicated that the strip was defective [23].

### 2.5. Data Analysis

Data were fed into the computer and analyzed using IBM SPSS software package version 20.0. (Armonk, NY, USA: IBM Corp). Categorical data were represented as numbers and percentages. The chi-square test was applied to investigate the association between the categorical variables. Alternatively, the Fisher Exact correction test was applied when more than 20% of the cells had an expected count less than 5. For continuous data, they were tested for normality using the Kolmogorov–Smirnov test. Quantitative data were expressed as range (minimum and maximum), mean, standard deviation, and median for normally distributed quantitative variables, and a Student’s *t*-test was used to compare two groups. On the other hand, for non-normally distributed quantitative variables, the Mann–Whitney test was used to compare two groups. Significance of the obtained results was judged at the 5% level.

## 3. Results

The prevalence of anti-SARS-CoV-2 IgM was 45.8% (n = 183) and of anti-SARS-CoV-2 IgG was 42.3% (n = 169). Seropositivity for both anti-SARS-CoV-2 IgM and IgG was 23.3% (n = 93), with statistically significant association (*p* < 0.001). The mean age of the 400 participants enrolled in the present study was 34.5 years (±13.5 SD). The mean age of the IgM positive participants (n = 183) was 33 years (±11.9 SD), (*p* = 0.033). The mean age of the IgG positive participants (n = 169) was 36.1 years (±15.5 SD), (*p* = 0.229). A total of 34.4 % (n = 63/183) and 49.1 % (n = 83/169) of the IgM positive and the IgG positive participants were in the age group ≥ 40 years, respectively.

Prior to testing for serology, most of the participants (59.5%; n = 238) did not do any previous RT-PCR testing for SARS-CoV-2-RNA. Significant correlations (*p* < 0.05) were found between the previous RT-PCR testing for SARS-CoV-2-RNA and anti-SARS-CoV-2 IgM and/or IgG positivity (Table 1). Among the participants that did not do any previous RT-PCR testing for SARS-CoV-2-RNA (n = 238), 50.0% (n = 119) were anti-SARS-CoV-2 IgM positive, and 26.1% (n = 62) were anti-SARS-CoV-2 IgG positive. Among the participants that were negative in their last two previous RT-PCR tests for SARS-CoV-2-RNA (n = 94), 40.4% (n = 38) were anti-SARS-CoV-2 IgM-positive, and 52.1% (n = 49) were anti-SARS-CoV-2 IgG-positive. On the other hand, among the participants that had at least one of their last two previous PCR tests positive for SARS-CoV-2-RNA (n = 68), 61.8% (n = 42) were anti-SARS-CoV-2 IgM-negative, and 14.7% (n = 10) were anti-SARS-CoV-2 IgG-negative cases. The mean interval between the date of the last previous PCR positive testing and date of immunoglobulins testing was 231.5 days (±71.9 SD).

Comparison of the history, demographics, anthropometrics, and lifestyle of the participants stratified for the extent of anti-SARS-CoV-2 IgM and IgG in their blood is summarized in Table 2 and Table 3, respectively. Males had higher seroprevalence for IgM and IgG than females (79.2 % and 81.7%, respectively) with a non-significant correlation between gender and each of IgM- or IgG-positivity.

Anti-SARS-CoV-2 IgM-positivity was more prevalent among students, smokers, and patients with chronic inflammatory and chronic respiratory diseases (*p* < 0.05). Anti-SARS-CoV-2 IgG-positivity was more prevalent among smokers, persons taking vitamin supplements, and patients with hypertension, type 2 diabetes, cardiovascular, and chronic inflammatory diseases (*p* < 0.05).

Although most of the participants (58.5%; n = 234/400) did not previously experience COVID-19-like symptoms, 49.1% (n = 115/234) and 25.6% (n = 60/234) of them were anti-SARS-CoV-2 IgM-positive and anti-SARS-CoV-2 IgG-positive, respectively. On the other hand, 41.5 % (n = 166/400) of the participants previously experienced symptoms similar to COVID-19, and among them there were anti-SARS-CoV-2 IgM-negative and IgG-negative cases at a prevalence of 59.0% (n = 98/166) and 34.3% (n = 57/166), respectively (*p* < 0.05). There was a predominance for the anti-SARS-CoV-2 IgM seronegativity and anti-SARS-CoV-2 IgG seropositivity among the participants that previously experienced symptoms similar to COVID-19.

Although 40.5% (n = 162/400) of the participants had encountered someone who was SARS-CoV-2 RT-PCR-positive, most of them were anti-SARS-CoV-2 IgM-negative at a prevalence of 72.2% (n = 117/162). On the other hand, most (58.6%; n = 95/162) of the participants, that had encountered someone who was SARS-CoV-2 RT-PCR-positive, were anti-SARS-CoV-2 IgG-positive (*p* < 0.05). Furthermore, 42.5% (n = 170/400) of the participants had encountered someone who was suffering from COVID-19-like symptoms but had no RT-PCR testing and/or had a negative RT-PCR test for SARS-CoV-2-RNA. Among them, 56.5% (n = 96/170) were anti-SARS-CoV-2 IgG-positive, whereas 73.5% (n = 125/170) of them were IgM-negative (*p* < 0.05).

At the time of the study, it is worth noting that the SARS-CoV-2 vaccination rate at our locality in Saudi Arabia was 43.8% (n = 175/400) with predominance of AstraZeneca (24.0%; n = 96/400), followed by Pfizer (19.8%; n = 79/400) vaccination. Among the participants, 22.0% (n = 88/400) received one dose and 21.8% (n = 87/400) received two doses of the anti-SARS-CoV-2 vaccine. The mean interval between the date of the last vaccine dose and date of immunoglobulin testing was 54.8 days (± 54.3 SD). 52.5% (n = 96/183) and 72.2% (n = 122/169) of the anti-SARS-CoV-2 IgM positive and anti-SARS-CoV-2 IgG positive participants were vaccinated, respectively. There was a statistically significant correlation (*p* < 0.001) between the vaccination and each of anti-SARS-CoV-2 IgM and IgG positivity, with more positivity among those receiving the second vaccine dose; 33.3% of the IgM positive group received two vaccine doses compared to 19.1% of the same group who received one vaccine dose, and, 43.8% of the IgG positive group received two vaccine doses compared to 28.4% of the same group who received one vaccine dose. The unvaccinated participants represented 56.2% (n = 225/400). A total of 47.5% (n = 87/183) and 27.8% (n = 47/169) of the anti-SARS-CoV-2 IgM-positive and anti-SARS-CoV-2 IgG-positive participants were unvaccinated, respectively.

## 4. Discussion

The world is still in a race to limit the impacts of SARS-CoV-2 on economies, health systems, and communities. Early testing and vaccination are key turning factors in controlling COVID-19. Regarding SARS-CoV-2 infection control, it is essential to know the proportion of the population that has been exposed, infected, or became immune. Understanding the humoral immune response to SARSCoV-2 and its vaccines is crucial for optimization of the SARSCoV-2 community vaccination programs.

A high seroprevalence of anti-SARS-CoV-2 IgM and IgG (45.8%, and 42.3%, respectively) was detected in our study. These results are close to those reported in an Iraqi study in which the seroprevalence of anti-SARS-CoV-2 IgM and IgG was 31.08 % and 26.58 %, respectively [29]. Lower rates of IgM and IgG seropositivity were also reported in Iran (22% and 33%, respectively) [30], USA (2.49% and 4.16%, respectively) [31], and Sweden (1.7% and 6.8%, respectively) [32]. The higher seropositivity in the current study could confirm the high exposure detected in our previous study in which the prevalence of anti-SARS-CoV-2 IgM was high (65%) in 300 non-vaccinated participants [33] and/or could be due the availability of the anti-SARS-CoV-2 vaccines at our locality and the good community turnout to vaccination (43.8%); 52.5% of the IgM positive were vaccinated compared to 47.5% of the same group who were unvaccinated, and, 72.2% of the IgG positive group were vaccinated compared to 27.8% of the same group were unvaccinated. Furthermore, the high IgM and IgG seroprevalence detected in the conducted study among smokers, participants aged ≥40 years, and patients with chronic diseases could reflect their more susceptibility to the SARS-CoV-2 infection and the special care needed for them to prevent COVID-19 complications.

Although 41.5% of the participants in the performed study previously experienced COVID-19-like symptoms, the anti-SARS-CoV-2 IgM and IgG seronegativity reached 59.0% and 34.3% among them, respectively (*p* < 0.05). These data were consistent with Iraqi and Iranian studies [29,34]. It is obvious from these results that humoral immune response was induced in some SARS-CoV-2-exposed persons only, and the lack of immunoglobulins in other cases may make them more susceptible to reinfection, especially with the emerging SARS-CoV-2 variants. We also noticed that 58.5% of the participants did not previously experience COVID-19-like symptoms, despite being seropositive for anti-SARS-CoV-2 IgM and IgG at 49.1% and 25.6% rates, respectively, with higher IgM/IgG seroprevalence among males. Those persons could be a dangerous source for SARS-CoV-2 transmission by acting as asymptomatic SARS-CoV-2 carriers. Lower rates of asymptomatic SARS-CoV-2 carriers were reported in Iraq, where anti-SARS-CoV-2 IgM and IgG were detected at 17.19%, and 16.56% rates, respectively [29]. Likewise, it was reported that 18.0% of the tested asymptomatic persons were seropositive for the SARS-CoV-2 in Iran [30]. A recent Saudi, multi-center, retrospective, cross-sectional study reported that around 9.3% (n = 142) of SARS-CoV-2 infected patients were asymptomatic, mainly in the 26–35 age group. Males represented 54.9% of the asymptomatic group. The study suspected that the percentage of the asymptomatic cases might be higher and attributed that to some clinical practices, such as taking swabs mainly from symptomatic patients in some centers, in the early phases of the pandemic [35]. On the other hand, a high rate of asymptomatic SARS-CoV-2 infected patients was reported in Japan (50.5% of cases were asymptomatic as of 20 February 2020) and this could be due to testing of more persons [36].

The proportion of undiagnosed asymptomatic/mild infections is a valuable quantity to measure the accurate burden of the disease and better predict its transmission potential. Consequently, population screening is mandatory for estimation of the asymptomatic proportion of SARS-CoV-2 carriage [37]. Moreover, the proportion of asymptomatic infection might be even higher since some cases may become negative upon RT–PCR testing. It was reported that seven SARS-CoV-2 patients, detected by using an antibody test, had negative RT–PCR results [38]. As a result, antibody testing could show a more accurate estimation of the asymptomatic SARS-CoV-2 carriers and the rate of disease exposure in general [39].

Prior to testing for immunoglobulins in the conducted study, although most of the participants (59.5%; n = 238/400) did not do any previous RT-PCR testing for SARS-CoV-2-RNA, there were positive cases for anti-SARS-CoV-2 IgM, and IgG among them at rates of 50.0% and 26.1%, respectively. The mean interval between the date of the last previous PCR positive test and date of immunoglobulins testing was 231.5 days (± 71.9 SD), which is more than enough for detection of immunoglobulins. Among the participants that were negative in the last two previous RT-PCR tests for SARS-CoV-2-RNA, 40.4 and 52.1% were positive for anti-SARS-CoV-2 IgM, and IgG, respectively. On the other hand, among the participants that have at least one of the last two previous PCR tests positive for SARS-CoV-2-RNA, 61.8, and 14.7% were negative for anti-SARS-CoV-2 IgM, and IgG, respectively. It is understandable from these results that the correlation between the previous RT-PCR results and the anti-SARS-CoV-2 IgM/IgG seropositivity is not uniform. Depending on the RT-PCR testing only for the diagnosis of SARS-CoV-2 infected cases does not reflect the true spread of COVID-19 in the community because of the high cost of the test, only clinically symptomatic cases are tested, and many clinically infected patients may give false negative results during their initial RT-PCR testing [40].

In March 2020, the FDA allowed lab manufacturers and researchers to promote serologic tests that met the certain criteria for accuracy and reliability without requiring full FDA approval. These serological tests were used on a large scale not only to reflect the real spread of the virus, but also to identify the potentially immune persons who might be “potentially protected” against re-infection and could provide potential sources of convalescent plasma for treatment of critical cases [41,42,43]. Therefore, antibody testing of random samples of the community can assess the progress towards immunization, vaccine efficiency, and efficiency of the implemented containment procedures, along with estimating the SARS-CoV-2 prevalence.

It is worth noting that the SARS-CoV-2 vaccination rate at our locality in Saudi Arabia, at the time of the study, was 43.8% with predominance of AstraZeneca ascribed to their safety, efficiency, affordability, and convenient storage and distribution [44]. However, many studies reported the higher efficiency of multiple mRNA vaccines, such as the Pfizer vaccine, predominantly prescribed nationally later in the disease path [45,46,47]. The current results showed significant correlation between the vaccination and anti-SARS-CoV-2 IgM and/or IgG positivity, with more positivity after receiving the second vaccine dose. Furthermore, the mean interval between the date of the last vaccine dose and date of immunoglobulin testing was 54.8 days. It is important to study the duration and degree of immunity generated after the vaccination and/or recovery from SARS-CoV-2 and its efficiency to protect against re-infection.

Several researchers studying the SARS-CoV-2 adaptive immune response reported that most SARS-CoV-2 convalescent persons have variable neutralizing antibody levels, depending on the numbers of SARS-CoV-2-specific T cells [48,49,50,51]. Recent research reported that the levels of IgG and neutralizing antibodies start to decrease within 2–3 months after recovery from COVID-19. Furthermore, the level of IgG in asymptomatic patients is lower than that in symptomatic cases and might disappear more rapidly [39]. Another study reported that two months after onset of illness, four (out of eight) COVID-19 convalescent cases showed decreased neutralizing antibodies [52]. In addition, a mathematical model predicts a short period of immunity after recovery from COVID-19 [53]. On the other hand, many recent research teams reported that the levels of antibodies progressively decrease after recovery from SARS-CoV-2 infection, but the immune memory persists for months, leading to more rapid and sustained immune response of the SARS-CoV-2-recovered individuals to the SARS-CoV-2 vaccines than the previously noninfected ones. Furthermore, they concluded that an effective immune response of the SARS-CoV-2-recovered persons can be achieved after a single dose of the vaccine [25,54,55,56,57,58,59,60,61].

Thus, according to the high rate of exposure (65%) to SARS-CoV-2 detected among non-vaccinated participants at our locality in our previous study [33], the high seroprevalence of anti-SARS-CoV-2 IgM and IgG (45.8%, and 42.3%, respectively), the percentage of unvaccinated anti-SARS-CoV-2 IgM and IgG positive participants (47.5 and 27.8% of the IgM positive and IgG positive participants were unvaccinated, respectively), and the high SARS-CoV-2 vaccination rate (43.8%) detected at our locality in the current study, these data can predict the success of the public health interventions towards efficient immunization of our community.

The limitations of our study include not testing the neutralizing potential of the detected antibodies, not determining the effects of SARS-CoV-2 variants, and the inability of serological testing to determine whether seropositivity is indicative of infection versus vaccination. However, we believe that our findings are useful and that they raise important questions that should be investigated in further studies regarding the role of the serological tests in the assessment of the real extent of SARS-CoV-2 exposure among the community, and the monitoring of the response and duration of SARS-CoV-2-antibody-mediated immunity either after infection or vaccination.

## 5. Conclusions and Recommendations

As far as we know, this is the first study to screen the seroprevalence of anti-SARS-CoV-2 IgM/IgG among inhabitants of Sakaka, Aljouf, Saudi Arabia, and it could be helpful for health policy makers. A high seroprevalence of anti-SARS-CoV-2 IgM and IgG positivity was detected especially among smokers, participants aged ≥40 years, and patients with chronic diseases, indicating their more susceptibility to the disease and the special care was needed for them to prevent COVID-19 complications. Our results revealed a high exposure of the community to SARS-CoV-2 with significant carrier proportion. Fortunately, the high percentage of IgM and IgG positivity among SARS-CoV-2 vaccinated individuals strengthens the potentially protective role of the vaccination. Our data support previous reports about the induction of a more rapid and sustained humoral immune response in the SARS-CoV-2-recovered individuals by the SARS-CoV-2 vaccines than the previously uninfected ones. Depending on RT-PCR testing only for the diagnosis of SARS-CoV-2 infected cases does not reflect the magnitude of COVID-19 in the community. Public assessment reflects the real scale of the disease exposure among the community and helps in identifying the asymptomatic carriers that constitute a major challenge in implementing effective SARS-CoV-2 prevention and control strategies. To limit the spread of the virus, rigorous implementation of a large-scale SARS-CoV-2 vaccination and anti-SARS-CoV-2 serological testing strategies should be empowered.

## Figures and Tables

**Table 1:** The previous PCR results of the participants stratified for expression of anti-SARS-CoV-2 IgM and/or IgG in their blood. Data shown are frequencies; n. (%), and mean ± SD (median; range).

		Total (n = 400)	IgM		Test of Significance	*p*	IgG		Test of Significance	*p*
			Negative n = 217 (54.2%)	Positive n = 183 (45.8%)			Negative n = 231 (57.7%)	Positive n = 169 (42.3%)		
Previous PCR testing	Not done	238 (59.5%)	119 (54.8%)	119 (65%)	χ2 = 12.326	0.006 *	176 (76.2%)	62 (36.7%)	χ2 = 82.708	<0.001 *
	Done and the last two PCR tests were positive	50 (12.5%)	36 (16.6%)	14 (7.7%)			5 (2.2%)	45 (26.6%)		
	Done and the last two PCR tests were negative	94 (23.5%)	56 (25.8%)	38 (20.8%)			45 (19.5%)	49 (29%)		
	Done and the last two PCR tests; one was positive and the other was negative	18 (4.5%)	6 (2.8%)	12 (6.6%)			5 (2.2%)	13 (7.7%)		
Interval between the date of the last PCR positive testing and date of Ig testing (days)	n.^≠^ (Mean ± SD.)	68 (231.5 ± 71.9)	42 (206.2 ± 47.5)	26 (272.5 ± 85.6)	*t* = 3.618 *	0.001 *	10 (214.8 ± 72.5)	58 (234.4 ± 72)	*t* = 0.795	0.430
	Median (Min.–Max.)	227.5 (78–385)	220.5 (78–308)	291 (105–385)			216 (138–385)	228.5 (78–383)		

Ig: Immunoglobulin. SD: Standard deviation. t: Student’s *t*-test. χ^2^: Chi-square test. *p*: *p* value for comparing between negative and positive IgM. ^≠^: The number of participants who are sure about the date of the last PCR positive testing. *: Statistically significant at *p* ≤ 0.05.

**Table 2:** Comparison of the history, demographics, anthropometrics, and lifestyle of the participants stratified for the extent of anti-SARS-CoV-2 IgM in their blood (Total n = 400). Data shown are frequencies; n. (%), and mean ± SD (median; range).

		Total (n = 400)	IgM		Test of Sig.	*p*
			Negative n = 217 (54.2%)	Positive n = 183 (45.8%)		
Age (years)	Mean ± SD.	34.5 ± 13.5	35.8 ± 14.6	33 ± 11.9	U = 17402.50 *	0.033 *
	Median (Min.–Max.)	35.5 (7–82)	39 (7–82)	33 (13–80)		
	˂20	61 (15.3%)	33 (15.2%)	28 (15.3%)	χ2 = 10.597 *	0.031 *
	20–29	91 (22.8%)	43 (19.8%)	48 (26.2%)		
	30–39	78 (19.5%)	34 (15.7%)	44 (24%)		
	40–49	125 (31.3%)	78 (35.9%)	47 (25.7%)		
	≥50	45 (11.3%)	29 (13.4%)	16 (8.7%)		
Gender	Male	311 (77.8%)	166 (76.5%)	145 (79.2%)	χ2 = 0.430	0.512
	Female	89 (22.3%)	51 (23.5%)	38 (20.8%)		
Education	Illiterate	74 (18.5%)	54 (24.9%)	20 (10.9%)	χ2 = 15.251 *	0.002 *
	Student	134 (33.5%)	64 (29.5%)	70 (38.3%)		
	Bachelor	109 (27.3%)	61 (28.1%)	48 (26.2%)		
	Postgraduate study	83 (20.8%)	38 (17.5%)	45 (24.6%)		
Occupation	Medical/Allied heath student	19 (4.8%)	7 (3.2%)	12 (6.6%)	χ2 = 2.542	0.281
	Healthcare Workers	62 (15.5%)	33 (15.2%)	29 (15.8%)		
	Others	319 (79.8%)	177 (81.6%)	142 (77.6%)		
Height (cm)	Mean ± SD.	172.3 ± 9.61	170.4 ± 11	174.5 ± 7	U = 15651.0 *	<0.001 *
	Median (Min.–Max.)	174 (90–193)	172 (90–191)	175 (155–193)		
Weight (kg)	Mean ± SD.	78.5 ± 13.1	78.8 ± 14.5	78.3 ± 11.2	*t* = 0.402	0.688
	Median (Min.–Max.)	80 (21–120)	80 (21–115)	79 (55–120)		
Lifestyle	Smoking	68 (17%)	20 (9.2%)	48 (26.2%)	χ2 = 20.365 *	<0.001 *
	Physical activity	177 (44.3%)	111 (51.2%)	66 (36.1%)	χ2 = 9.159 *	0.002 *
	Healthy diet	204 (51%)	132 (60.8%)	72 (39.3%)	χ2 = 18.339 *	<0.001 *
	Vitamin supplements	118 (29.5%)	56 (25.8%)	62 (33.9%)	χ2 = 3.111	0.078
	Herbal preferences	2 (0.5%)	0 (0%)	2 (1.1%)	χ2 = 2.384	^FE^*p* = 0.209
Comorbidities	Hypertension	46 (11.5%)	22 (10.1%)	24 (13.1%)	χ2 = 0.864	0.353
	Diabetes Type 2	29 (7.3%)	19 (8.8%)	10 (5.5%)	χ2 = 1.599	0.206
	Diabetes Type 1	3 (0.8%)	0 (0%)	3 (1.6%)	χ2 = 3.584	^FE^*p* = 0.095
	CVD	12 (3%)	8 (3.7%)	4 (2.2%)	χ2 = 0.768	0.381
	Kidney	3 (0.8%)	0 (0%)	3 (1.6%)	χ2 = 3.584	^FE^*p* = 0.095
	GERD	23 (5.8%)	21 (9.7%)	2 (1.1%)	χ2 = 13.50 *	<0.001 *
	Autoimmune	2 (0.5%)	0 (0%)	2 (1.1%)	χ2 = 2.384	^FE^*p* = 0.209
	Chronic inflammatory	5 (1.3%)	0 (0%)	5 (2.7%)	χ2 = 6.004	0.019 *
	Chronic respiratory	19 (4.8%)	3 (1.6%)	16 (7.4%)	χ2 = 7.214 *	0.007 *
Type of the current medications	Antihypertensive	41 (10.3%)	18 (8.3%)	23 (12.6%)	χ2 = 1.971	0.160
	Anti-diabetic	33 (8.3%)	19 (8.8%)	14 (7.7%)	χ2 = 0.160	0.689
	Antibiotic	6 (1.5%)	1 (0.5%)	5 (2.7%)	χ2 = 3.467	^FE^*p* = 0.098
	Anti-inflammatory	5 (1.3%)	0 (0%)	5 (2.7%)	χ2 = 6.004	^FE^*p* = 0.019 *
	Immunosuppressive	2 (0.5%)	0 (0%)	2 (1.1%)	χ2 = 2.384	^FE^*p* = 0.209
	Blood thinners	8 (2%)	8 (3.7%)	0 (0%)	χ2 = 6.884	^FE^*p* = 0.009 *
Previous COVID-19-like symptoms	No	234 (58.5%)	119 (54.8%)	115 (62.8%)	χ2 = 2.619	0.106
	Yes	166 (41.5%)	98 (45.2%)	68 (37.2%)		
IgG	Negative	231 (57.8%)	141 (65%)	90 (49.2%)	χ2 = 10.153 *	0.001 *
	Positive	169 (42.3%)	76 (35%)	93 (50.8%)		
Contact with a person having a positive SARS-CoV-2-RNA PCR test	No	238 (59.5%)	100 (46.1%)	138 (75.4%)	χ2 = 35.433 *	<0.001 *
	Yes	162 (40.5%)	117 (53.9%)	45 (24.6%)		
Contact with a person suffering from COVID-19-like symptoms without or with negative RT-PCR results	No	230 (57.5%)	92 (42.4%)	138 (75.4%)	χ2 = 44.277 *	<0.001 *
	Yes	170 (42.5%)	125 (57.6%)	45 (24.6%)		
Vaccination	No	225 (56.2%)	138 (63.6%)	87 (47.5%)	χ2 = 10.397 *	0.001*
	Yes	175 (43.8%)	79 (36.4%)	96 (52.5%)		
	AstraZeneca	96 (24%)	54 (24.9%)	42 (23%)	χ2 = 0.204	0.652
	Pfizer	79 (19.8%)	25 (11.5%)	54 (29.5%)	χ2 = 20.266 *	<0.001 *
Doses of vaccines	No dose	225 (56.3%)	138 (63.6%)	87 (47.5%)	χ2 = 26.625	<0.001 *
	One dose only	88 (22%)	53 (24.4%)	35 (19.1%)		
	Two doses	87 (21.8%)	26 (12%)	61 (33.3%)		
Interval between the date of first vaccine dose and date of Ig testing (days)	n.≠ (Mean ± SD.)	175 (83.8 ± 73.7)	79 (61.9 ± 70.3)	96 (101.8 ± 71.9)	U = 2429.50	<0.001 *
	Median (Min.–Max.)	42 (0–251)	27 (0–251)	99.5 (5–241)		
Interval between the date of second vaccine dose and date of Ig testing (days)	n.≠ (Mean ± SD.)	87 (77.3 ± 57.9)	26 (78.3 ± 51.1)	61 (76.9 ± 60.9)	U = 771.0	0.838
	Median (Min.–Max.)	71 (1–194)	87 (2–150)	62 (1–194)		
Interval between the date of last vaccine dose and date of Ig testing (days)	n.^≠^ (Mean ± SD.)	175 (54.8 ± 54.3)	79 (43.2 ± 44.1)	96 (64.4 ± 59.9)	U = 2951.50	0.012 *
	Median (Min.–Max.)	29 (0–241)	23 (0–182)	32.5 (1–241)		

CVD: Cardiovascular disease. FE: Fisher Exact. GERD: Gastroesophageal reflux disease. Ig: Immunoglobulin. SD: Standard deviation. T: Student’s *t*-test. U: Mann–Whitney test. χ^2^: Chi-square test. *p*: *p* value for comparing between negative and positive IgM. ^≠^: The number of participants who are sure about the date of vaccination. *: Statistically significant at *p* ≤ 0.05.

**Table 3:** Comparison of the history, demographics, anthropometrics, and lifestyle of the participants stratified for the extent of anti-SARS-CoV-2 IgG in their blood (Total n = 400). Data shown are frequencies; n. (%), and mean ± SD (median; range).

		Total (n = 400)	IgG		Test of Sig.	*p*
			Negative n = 231 (57.7%)	Positive n = 169 (42.3%)		
Age (years)	Mean ± SD.	34.5 ± 13.5	33.4 ± 11.7	36.1 ± 15.5	U = 18148	0.229
	Median (Min.–Max.)	35.5 (7–82)	34 (7–60)	39 (10–82)		
	˂20	61 (15.3%)	33 (14.3%)	28 (16.6%)	χ2 = 8.842	0.065
	20-29	91 (22.8%)	60 (26%)	31 (18.3%)		
	30-39	78 (19.5%)	51 (22.1%)	27 (16%)		
	40-49	125 (31.3%)	67 (29%)	58 (34.3%)		
	≥50	45 (11.3%)	20 (8.7%)	25 (14.8%)		
Gender	Male	311 (77.8%)	173 (74.9%)	138 (81.7%)	χ2 = 2.582	0.108
	Female	89 (22.3%)	58 (25.1%)	31 (18.3%)		
Education	Illiterate	74 (18.5%)	43 (18.6%)	31 (18.3%)	χ2 = 0.819	0.845
	Student	134 (33.5%)	74 (32%)	60 (35.5%)		
	Bachelor	109 (27.3%)	63 (27.3%)	46 (27.2%)		
	Postgraduate study	83 (20.8%)	51 (22.1%)	32 (18.9%)		
Occupation	Medical/Allied heath student	19 (4.8%)	9 (3.9%)	10 (5.9%)	χ2 = 1.238	0.538
	Healthcare Workers	62 (15.5%)	34 (14.7%)	28 (16.6%)		
	Others	319 (79.8%)	188 (81.4%)	131 (77.5%)		
Height (cm)	Mean ± SD.	172.3 ± 9.61	170.2 ± 9.1	175.1 ± 9.6	U = 12133.0 *	<0.001 *
	Median (Min.–Max.)	174 (90–193)	172 (90–190)	177 (135–193)		
Weight (kg)	Mean ± SD.	78.5 ± 13.1	76.3 ± 12.4	81.5 ± 13.5	*t* = 3.997 *	<0.001 *
	Median (Min.–Max.)	80 (21–120)	79 (21–120)	80 (38–120)		
Lifestyle	Smoking	68 (17%)	24 (10.4%)	44 (26%)	χ2 = 16.932 *	<0.001 *
	Physical activity	177 (44.3%)	98 (42.4%)	79 (46.7%)	χ2 = 0.739	0.390
	Healthy diet	204 (51%)	113 (48.9%)	91 (53.8%)	χ2 = 0.949	0.330
	Vitamin supplements	118 (29.5%)	48 (20.8%)	70 (41.4%)	χ2 = 19.993 *	<0.001 *
	Herbal preferences	2 (0.5%)	1 (0.4%)	1 (0.6%)	χ2 = 0.049	^FE^*p* = 1.000
Comorbidities	Hypertension	46 (11.5%)	9 (3.9%)	37 (21.9%)	χ2 = 31.061 *	<0.001 *
	Diabetes Type 2	29 (7.3%)	7 (3%)	22 (13%)	χ2 = 14.478 *	<0.001 *
	Diabetes Type 1	3 (0.8%)	1 (0.4%)	2 (1.2%)	χ2 = 0.739	^FE^*p* = 0.576
	CVD	12 (3%)	1 (0.4%)	11 (6.5%)	χ2 = 12.382 *	<0.001 *
	Kidney	3 (0.8%)	1 (0.4%)	2 (1.2%)	χ2 = 0.739	^FE^*p* = 0.576
	GERD	23 (5.8%)	10 (4.3%)	13 (7.7%)	χ2 = 2.037	0.153
	Autoimmune	2 (0.5%)	1 (0.4%)	1 (0.6%)	χ2 = 0.049	^FE^*p* = 1.000
	Chronic inflammatory	5 (1.3%)	0 (0%)	5 (3%)	χ2 = 6.921	^FE^*p* = 0.013 *
	Chronic respiratory	19 (4.8%)	6 (3.6%)	13 (5.6%)	χ2 = 0.931	0.335
Type of the current medications	Antihypertensive	41 (10.3%)	6 (2.6%)	35 (20.7%)	χ2 = 34.805	<0.001 *
	Anti-diabetic	33 (8.3%)	8 (3.5%)	25 (14.8%)	χ2 = 16.551	<0.001 *
	Antibiotic	6 (1.5%)	2 (0.9%)	4 (2.4%)	χ2 = 1.488	^FE^*p* = 0.246
	Anti-inflammatory	5 (1.3%)	3 (1.3%)	2 (1.2%)	χ2 = 0.011	^FE^*p* = 1.000
	Immunosuppressive	2 (0.5%)	0 (0%)	2 (1.2%)	χ2 = 2.747	^FE^*p* = 0.178
	Blood thinners	8 (2%)	0 (0%)	8 (4.7%)	χ2 = 11.158	^FE^*p* = 0.001 *
Previous COVID-19-like symptoms	No	234 (58.5%)	174 (75.3%)	60 (35.5%)	χ2 = 63.749 *	<0.001 *
	Yes	166 (41.5%)	57 (24.7%)	109 (64.5%)		
IgM	Negative	217 (54.3%)	141 (61%)	76 (45%)	χ2 = 10.153*	0.001 *
	Positive	183 (45.8%)	90 (39%)	93 (55%)		
Contact with a person having a positive SARS-CoV-2-RNA PCR test	No	238 (59.5%)	164 (71%)	74 (43.8%)	χ2 = 29.983 *	<0.001*
	Yes	162 (40.5%)	67 (29%)	95 (56.2%)		
Contact with a person suffering from COVID-19-like symptoms without or with negative RT-PCR results	No	230 (57.5%)	157 (68%)	73 (43.2%)	χ2 = 24.504 *	<0.001 *
	Yes	170 (42.5%)	74 (32%)	96 (56.8%)		
Vaccination	No	225 (56.2%)	178 (77.1%)	47 (27.8%)	χ2 = 96.177 *	<0.001 *
	Yes	175 (43.8%)	53 (22.9%)	122 (72.2%)		
	AstraZeneca	96 (24%)	32 (13.9%)	64 (37.9%)	χ2 = 30.864 *	<0.001 *
	Pfizer	79 (19.8%)	21 (9.1%)	58 (34.3%)	χ2 = 39.193 *	<0.001 *
Doses of vaccines	No dose	225 (56.3%)	178 (77.1%)	47 (27.8%)	χ2 = 112.870 *	<0.001 *
	One dose only	88 (22%)	40 (17.3%)	48 (28.4%)		
	Two doses	87 (21.8%)	13 (5.6%)	74 (43.8%)		
Interval between the date of first vaccine dose and date of Ig testing (days)	n.^≠^ (Mean ± SD.)	175 (83.8 ± 73.7)	53 (35.3 ± 39.2)	122 (104.8 ± 75.4)	U = 1480.5 *	<0.001 *
	Median (Min.–Max.)	42 (0–251)	24 (0–200)	107.5 (0–251)		
Interval between the date of second vaccine dose and date of Ig testing (days)	n.^≠^ (Mean ± SD.)	87 (77.3 ± 57.9)	13 (40.9 ± 51.7)	74 (83.7 ± 56.8)	U = 249.50 *	0.006 *
	Median (Min.–Max.)	71 (1–194)	23 (2–168)	82.5 (1–194)		
Interval between the date of last vaccine dose and date of Ig testing (days)	n.^≠^ (Mean ± SD.)	175 (54.8 ± 54.3)	53 (27.9 ± 32.8)	122 (66.5 ± 57.6)	U = 1842.50	<0.001 *
	Median (Min.–Max.)	29 (0–241)	21 (0–168)	40 (0–241)		

CVD: Cardiovascular disease. FE: Fisher Exact. GERD: Gastroesophageal reflux disease. Ig: Immunoglobulin. SD: Standard deviation. T: Student’s *t*-test. U: Mann–Whitney test. χ^2^: Chi-square test. *p*: *p* value for comparing between negative and positive IgG. ^≠^: The number of participants who are sure about the date of vaccination. *: Statistically significant at *p* ≤ 0.05.

## Data Availability

All data are available in the manuscript.
